# Subcomponent Vaccine Based on CTA1-DD Adjuvant with Incorporated UreB Class II Peptides Stimulates Protective *Helicobacter pylori* Immunity

**DOI:** 10.1371/journal.pone.0083321

**Published:** 2013-12-31

**Authors:** John G. Nedrud, Nayer Bagheri, Karin Schön, Wei Xin, Hilda Bergroth, Dubravka Grdic Eliasson, Nils Y. Lycke

**Affiliations:** 1 Department of Pathology, Case Western Reserve University, Cleveland, Ohio, United States of America; 2 Mucosal Immunobiology and Vaccine Research Center (MIVAC) and the Department of Microbiolgy and Immunology, Institute of Biomedicine, University of Gothenburg, Göteborg, Sweden; Instituto Butantan, Brazil

## Abstract

A mucosal vaccine against *Helicobacter pylori* infection could help prevent gastric cancers and peptic ulcers. While previous attempts to develop such a vaccine have largely failed because of the requirement for safe and effective adjuvants or large amounts of well defined antigens, we have taken a unique approach to combining our strong mucosal CTA1-DD adjuvant with selected peptides from urease B (UreB). The protective efficacy of the selected peptides together with cholera toxin (CT) was first confirmed. However, CT is a strong adjuvant that unfortunately is precluded from clinical use because of its toxicity. To circumvent this problem we have developed a derivative of CT, the CTA1-DD adjuvant, that has been found safe in non-human primates and equally effective compared to CT when used intranasally. We genetically fused the selected peptides into the CTA1-DD plasmid and found after intranasal immunizations of Balb/c mice using purified CTA1-DD with 3 copies of an *H. pylori* urease T cell epitope (CTA1-UreB3T-DD) that significant protection was stimulated against a live challenge infection. Protection was, however, weaker than with the gold standard, bacterial lysate+CT, but considering that we only used a single epitope in nanomolar amounts the results convey optimism. Protection was associated with enhanced Th1 and Th17 immunity, but immunizations in IL-17A-deficient mice revealed that IL-17 may not be essential for protection. Taken together, we have provided evidence for the rational design of an effective mucosal subcomponent vaccine against *H. pylori* infection based on well selected protective epitopes from relevant antigens incorporated into the CTA1-DD adjuvant platform.

## Introduction

*Helicobacter pylori* is a gram negative microaerophilic bacterium which infects the gastric mucosa of approximately half of the world's population and is a risk factor for both peptic ulcer disease and gastric cancers [1], [2]. The bacteria live in the mucus layer overlying gastric epithelial cells, an environment from which it is able to provoke host inflammatory and immune responses. These host responses are unable to eradicate the infection, however, so that without treatment, the infection can persist for decades or even the life of the host. Although pharmacologic agents can cure the infection, multi-drug regimens which can have significant side effects are required. Using combinations of antibiotics and agents such as proton pump inhibitors it is possible to achieve eradication rates as high as 80–90%, but failures can lead to antibiotic resistance and re-infection is not uncommon [3], [4]. An alternative and more attractive approach is vaccination which not only leads to more vigorous immune responses than infection but it is also likely to provide herd immunity, dramatically reducing spread of infection. Several candidate vaccines and mucosal vaccines, in particular, have been shown in animal models to reduce or eliminate bacterial load and disease in the stomach [5], [6].

Although an abundance of purified/recombinant *H. pylori* antigens and vaccine adjuvants have been successfully used in animal models of *H. pylori* vaccination, bacterial lysates and whole cell vaccines combined with the holotoxins cholera toxin (CT) or the closely related *E. coli* heat labile toxin (LT) as mucosal adjuvants have been the gold standard in animal models of *H. pylori* vaccination [5]. Most vaccine regimens require an adjuvant and work best intranasally (i.n) [7] or sublingually [8]. Numerous studies in animal models have also demonstrated that antibodies are not required for (but may participate in or even impair) protective immunity, but, in contrast, specific CD4 T cell responses are required for *H. pylori* vaccine efficacy [9], [10], [11], [12], [13]. Among subunit and vector vaccines, *H. pylori* urease has been a leading candidate [14], [15], [16] and both CD4 T cell and B cell peptide epitopes have been defined [17], [18].

Cholera toxin or LT have been the most effective and widely used adjuvants for *H. pylori* mucosal vaccines in animal models. These bacterial toxins are well tolerated when used at adjuvant effective doses in mice and other small animal models of *H. pylori* infection. CT and LT are too toxic for humans, however, and in a human clinical *H. pylori* vaccine trial, the use of holotoxin LT resulted in significant diarrhea in 2/3 of the vaccine recipients [19]. Mutations targeting the active sites of these molecules can reduce the toxicity while retaining adjuvant function and these mutant toxins have been used with some success as mucosal adjuvants for *H. pylori* vaccines [20], [21]. Our approach has been to create chimeric CT-derived molecules which retain the full enzymatic activity of the holotoxin, but which specifically target immune cells instead of all nucleated GM1-receptor carrying cells, including nerve cells [22]. In this approach we have linked the enzymatically active CTA1 fragment of CT to two copies of the D-fragment of *Staphylococcus aureus* protein A, a strong immunoglobulin binding domain, to create an adjuvant that we have named CTA1-DD [23]. We have shown that this molecule is non-toxic when delivered i.n to mice and non-human primates [24], [25] and significantly reduces the bacterial burden when used as a mucosal adjuvant for an *H. pylori* lysate vaccine in mice [26], [27]. We recently demonstrated a related approach in which a peptide from influenza virus, the M2e peptide, was inserted into CTA1-DD (CTA1-M2e-DD) and found to effectively protect against infection in mice [28], [29]. We now report that an MHC class II restricted *H. pylori* peptide inserted into CTA1-DD also can successfully immunize and protect Balb/c mice against *H. pylori* infection [18].

## Materials and Methods

### *H. pylori* growth, mice, immunization, and challenge

*H. pylori* for infection and preparation of bacterial lysate was grown as previously published [30]. Balb/c female mice aged 6–8 weeks were obtained from Charles River Laboratories (Portage, MI) or from Harlan Laboratories (the Netherlands) and were housed in microisolater cages under specific pathogen free (spf) conditions with free access to autoclaved food and water. IL-17 KO mice [31] on a Balb/c background, were bred in ventilated cages and kept together with the Balb/c wild-type mice under spf conditions at the University of Gothenburg Animal Facility (Gothenburg, Sweden). All mice infected with *H. pylori* were housed in A-BSL-2 rooms. Mice were immunized intranasally (i.n) while fully awake by administering 100 µg of *H. pylori* lysate plus 5 µg of CT adjuvant, 100 µg of *H. pylori* peptides plus 5 µg of CT adjuvant or 5 µg of CTA1-peptide-DD in 20 µl of saline, 10 µl at a time slowly to each nare. Immunizations were done as a single immunization or as series of 4 immunizations, once a week, as indicated in the figure legends. Immunized mice were challenged twice over three days with ca 10^7^ cfu of *H. pylori* strain SS-1 [32] in 0.5 ml of brucella broth at 4 weeks after completion of immunizations using flexible polyethylene tubing on the end of a tuberculin syringe. At 3 or 7 weeks after challenge mice were killed and stomachs were removed aseptically. These time intervals post-challenge were chosen based upon our previously published work showing a stable level of gastric *H. pylori* colonizing bacteria in non-immunized challenged mice and a consistent drop in the bacterial load from intranasally immunized mice over this time period [33], [34]. These published results were in C57BL/6 mice, but we have observed essentially identical kinetics for the drop in *H. pylori* colonizing bacteria also in immunized BALB/c mice, the mouse strain used in the present research (JGN, unpublished results). Noteworthy, protection induced by CTA1-UreB3T-DD immunizations was similar irrespective if we assessed it at 3 or 7 weeks after immunizations (see Results). A strip of stomach including both antrum and body was removed from the greater curvature and used for quantitative culture [33]. Additional sections of stomach were fixed in buffered formalin, embedded in paraffin, and sections were stained with H&E for scoring of gastric inflammation. Each experiment included challenge of both immunized and the internal control of non-immunized (administered saline) mice and most experiments also included the positive control of mice immunized with *H. pylori* lysate plus CT.

Ethics statement:All mouse protocols were approved by the Case Western Reserve University IACUC protocol number 09-0111 and the Ethical committee, the Board of Agriculture in Gothenburg, Sweden, ethical protocol number 60/11.

### *H. pylori* peptides

For immunizations, a linear B cell epitope peptide and two CD4 T cell epitope peptides were produced by Genscript (Piscataway, NJ) at >95% purity. The 19 amino acid ureB linear B cell epitope ureB321–339 (CHHLDKSIKEDVQFADSRI) represents a site which a urease neutralizing monoclonal antibody recognizes [17] and a fusion protein between this epitope and CTB as been shown to reduce *H. pylori* colonization when used as an oral vaccine [35]. The two CD4 T cell epitopes, 15 amino acid ureB237–251 (VADKYDVQVAIHTDT) (I-A^d^ restricted) and 16 amino acid ureB546–561 (FVDGKEVTSKPANKVS) (I-E^d^ restricted) [18] have been shown to act synergistically to induce T cell proliferation after subcutaneous immunization with CFA/IFA in BALB/c mice [18]. For peptide immunization of Balb/c mice, 100 µg of peptide was mixed with 5 µg of holotoxin CT (List Biological Laboratories, Campell, CA) in a total of 20 µl of PBS and 10 µl was administered i.n to each nare. When a combination of two different peptides were used for immunization, 50 µg of each peptide (100 µg total) were admixed with CT adjuvant, as described above.

### Genetic constructs incorporating *H. pylori* peptides into CTA1-DD fusion proteins

CTA1-DD fusion protein alone or CTA1-DD constructions containing a single copy or 3 copies of UreB_237–251_ peptide, CTA1-UreB3T-DD, or three copies of the influenza virus antigen M2e (amino acid sequence VADKYDVQVAIHTDT), were produced in *Escherichia coli* as previously described [23]. Another fusion protein containing single copies of the CD4 T cell epitope peptide UreB_237–251_ plus the B cell epitope peptide UreB_321—339_ incorporated into the CTA1-DD backbone was produced in a similar manner. ADP-ribosyltransferase enzymatic activity was tested using the NAD:agmatine assay as described previously [36]. Protein analysis was performed with SDS-PAGE, and concentrations were determined using the Bio-Rad DC protein assay, according to the manufacturer's instructions. Endotoxin levels were assessed by the *Limulus* amebocyte lysate test (LAL Endochrome; Charles River Endostestafe). The endotoxin levels were <1 EU/mg protein in all preparations used.

### Determinations of antibodies, cytokines and cell proliferation

Serum IgG anti-H. pylori antibodies were analyzed by ELISA as previously described using *H. pylori* lysate (10 µg/ml) as the coating antigen [33]. At the time of harvest, spleens were removed and cultured for 48–72 hr together with recall antigen in RPMI media or Iscove's media (IMDM, HyClone), supplemented with 10% fetal bovine serum, 5×10^−5^ M beta mercaptoethanol and antibiotics at 2×10^6^ cells/ml. For antigen recall responses we used *H. pylori* lysate (10 µg/ml), recombinant UreB (rUreB) (10 µg/ml) (kind gift from C. Flach and J. Holmgren, University of Gothenburg, Sweden), CTA1-UreB3T-DD (1 µM), CTA1-DD (1 µM) or purified peptides, UreB T 237–251 and UreB B 321–339, Innovagen AB (Lund, Sweden), at 5 µg/ml or as specified in the figure legends and text describing each experiment. To assess UreB-specific CD4 T cell responses we enriched splenocytes from immunized mice with AutoMacs (Miltenyi Biotech, Köln, Germany) (purity >96%) and cultured these cells at 100.000 cells/well together with irradiated splenocytes from nu/nu Balb/c mice and recall antigen (UreB and CTA1-Ureb3T-DD) as described above. CD4 T cell antigen-recall responses were analyzed in culture supernates for IFNγ, IL-17A, IL-17F and other cytokines as indicated using specific Duoset kits (R&D Systems, Minneapolis, MN) according to manufacture's instructions. Specific cell proliferation to recall antigen was assessed after 72 h of culture, by the addition of 1 µCi/well ^3^H-thymidine (PerkinElmer, USA) for the last 6 hours of culture. The ^3^H-thymidine uptake was determined using a scintillation counter (Beckman, LKB, Bromma, Sweden). Data were expressed as mean c.p.m ± S.E.M. of 5 mice per group in each experiment.

### Statistical analysis

The parametric ANOVA analysis with post-hoc Bonferroni tests for multiple groups was used for analysis of statistical significance in all experimental groups. Statistical significance is also given as p-values: ***p<0.001, **p<0.01 and *p<0.05.

## Results

**Figure 1:**
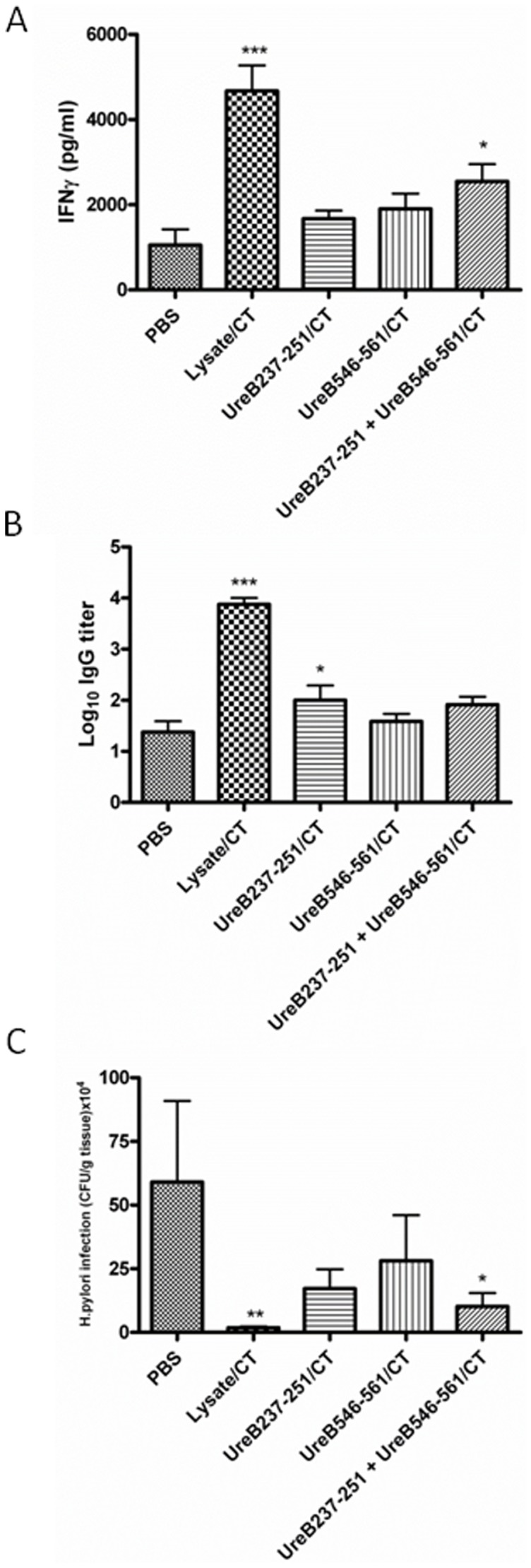
Groups of 6 Balb/c mice were left unimmunized (PBS) or were immunized intranasally 4 times with 100 µg of *H. pylori* lysate+5 µg of CT adjuvant, 100 µg of Hp UreB237–251+5 µg of CT adjuvant, 100 µg of Hp UreB546–561+5 µg of CT adjuvant or a mixture of the two peptides (50 µg each)+5 µg of CT adjuvant, as described in the Methods section. Four weeks after completion of immunizations all mice were challenged twice over three days with ca 2×10^7^ cfu of *H. pylori* SS-1 and **3** weeks later mice were killed and recall splenic IFNγ responses in supernatants from lysate stimulated cultures with unstimulated background controls subtracted (A), serum IgG anti-*H. pylori* antibody responses (B) and gastric *H. pylori* cfu/g of stomach tissue (C) were determined. These are representive data from at least 3 identical experiments giving similar results. ANOVA was used for statistical analysis: * p<0.05, ** p<0.01 and ***p<0.001.

We first confirmed the capacity of two different *H. pylori* urease B subunit MHC class II restricted peptides, UreB237–251 and UreB546–561, to prime for T cell and antibody responses following i.n immunizations of Balb/c mice using CT adjuvant and challenging with live bacteria. Panel A of Figure 1 shows that after *H. pylori* challenge of mice previously immunized with a mixture of the two peptides, a significantly elevated IFNγ response was observed after *in vitro* recall stimulation with *H. pylori* lysate (P = 0.014). There was also a trend towards elevated responses after immunization with the individual peptides. However, these results did not reach statistical significance. In a similar manner, Figure 1B shows that elevated levels of serum IgG antibody versus *H. pylori* lysate was observed when mice were immunized with peptide UreB237–251 (P = 0.026), but not with UreB546–561 or a mixture of the two peptides. Most importantly, the capacity of the T cell epitopes to prime for anti-*H. pylori* immune responses was also shown to have functional activity since mice immunized i.n with a mixture of the two peptides plus CT adjuvant exhibited a 5.5 fold reduction in gastric *H. pylori* (p = 0.050) (Figure 1C). This reduction in bacterial load was not as great as the 27-fold reduction (p = 0.022) observed after mice were immunized with lysate plus CT, however. There was a positive correlation between protective efficacy and increased priming of IFNγ producing spleen T cells.

**Figure 2:**
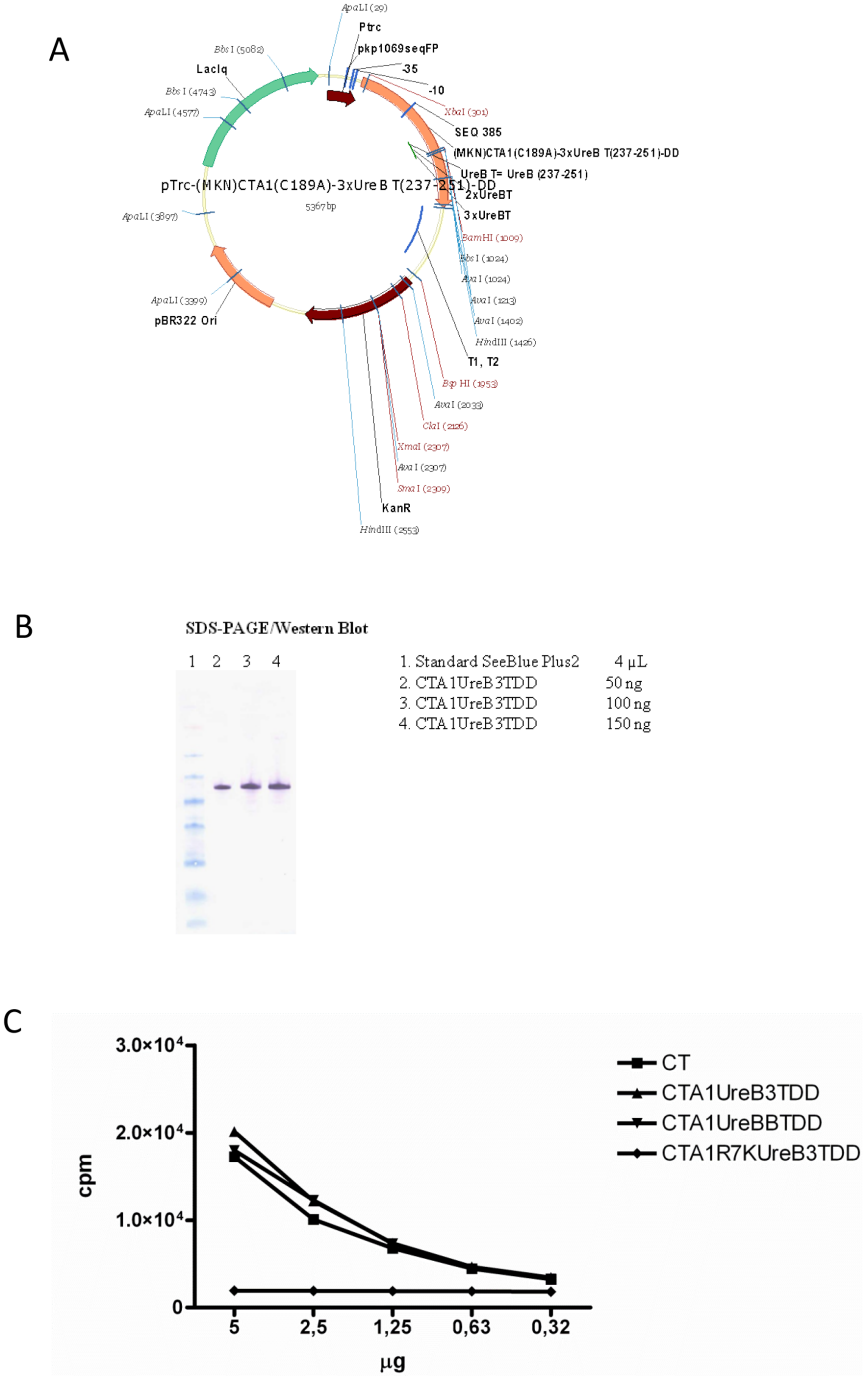
Schematic drawing of the CTA1-DD plasmid vector with incorporated 1–3 UreB 237–251 epitopes in single or tandem repeats. (A). Protein analysis was performed with SDS-PAGE on purified material from the *E.coli* expression system and concentrations were determined using the Bio-Rad DC protein assay, according to the manufacturer's instructions. Western blot analysis using HRP-labelled anti-DD antibodies (B). The ADP-ribosylating ability of CTA1-UreB3T-DD, but not the mutant inactive CTA1R7K-UreB3T-DD protein, was comparable to that of CT at a molar level, as shown in the NAD-agmatine assay diagram (C). These are representive data from at least 5 identical experiments giving similar results.

**Figure 3:**
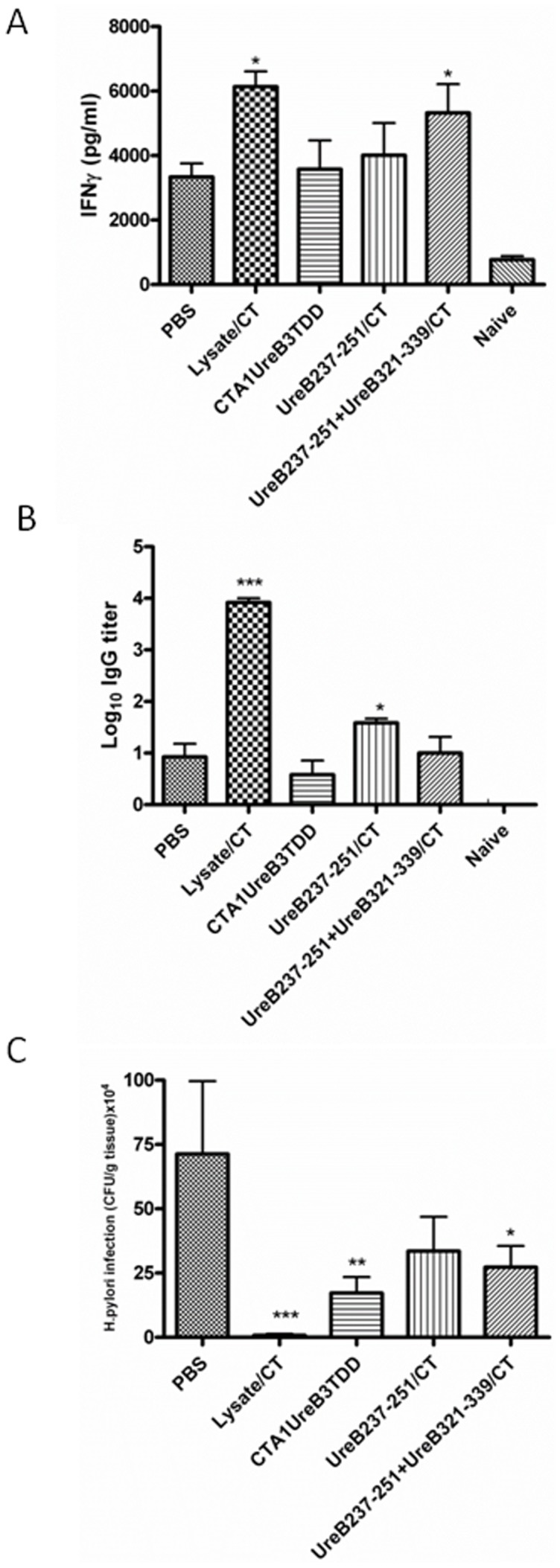
Groups of 6 or 7 Balb/c mice were left unimmunized (PBS) or were immunized intranasally 4 times with *H. pylori* lysate+5 µg of CT adjuvant, a fusion protein of CTA1-UreB3T-DD containing three copies of UreB237–251, UreB237–251 peptide alone+5 µg of CT adjuvant, or a mixture of UreB237–251 plus the UreB B321–339 peptide+5 µg of CT adjuvant, as described in Fig. 1. Four weeks after completion of immunization a separate group of 3 naïve mice were left unchallenged (Naïve) and all other mice were challenged twice over three days with ca 2×10^7^ cfu of *H. pylori* SS-1 and 3 weeks later mice were killed and recall splenic IFNγ responses in supernatants from lysate stimulated cultures with unstimulated background controls subtracted (A), serum IgG anti-*H. pylori* antibody responses (B) and gastric *H. pylori* cfu/g of stomach tissue (C) were determined. For IFNγ determinations (A) only one experiment with CTA1-UreB3T-DD was included, but apart from that these are representive data from at least 3 identical experiments giving similar results. ANOVA was used for statistical analysis :* p<0.05, ** p<0.01 and ***p<0.001.

Based upon the capacity of these *H. pylori* peptides to induce anti-*H. pylori* immunity following immunization, we elected to generate a genetic fusion protein construct incorporating 3 copies of UreB237–251 within CTA1-DD, CTA1-UreB3T-DD, as shown in Figure 2A and described under Methods. The resulting fusion protein was expressed and purified as described [23] and Figure 2B shows a western blot of the purified fusion protein probed with an anti-DD monoclonal antibody [23]. We also demonstrated that the fusion protein possessed full ADP-ribosylation activity (a measure of preserved enzymatic function of the CTA1 moiety) comparable to that of native CT (Figure 2C). Next we evaluated this fusion protein as an i.n *H. pylori* vaccine and compared it with i.n *H. pylori* lysate plus CT, UreB237–251 plus CT, or a mixture of UreB237–251 and a B cell epitope peptide, UreB321–339, plus CT. As shown in Figure 3, lysate significantly primed IFNγ producing CD4 T cells as did the mixture of UreB237–251 and UreB321–339 (p<0.05)(Fig. 3A). However, similar to the results in Figure 1 only lysate and UreB237–251 plus CT adjuvant yielded enhanced antibody responses (p<0.001 and p<0.05, respectively) (Fig. 3B). When the bacterial load was examined after challenge of mice immunized i.n with CTA1-UreB3T-DD significant reduction in cfu was achieved (p = 0.02, 4-fold reduction), although this effect was not as great as that achieved by lysate plus CT (p = 0.004, 90-fold reduction) (Fig. 3C). Noteworthy, the dose of peptide incorporated in the fusion protein was in the 1 nanomolar range, whereas the dose of UreB237–251 (p = 0.10, 2-fold reduction) or a mixture of UreB237–251 and UreB321–339 plus CT adjuvant (p = 0.06, 3-fold reduction) was 60-fold higher, but still CTA1-UreB3T-DD resulted in lower cfu and stronger protection (Fig. 3C). Since we and others have observed enhanced gastritis in protectively immunized mice [33], [34], we also examined gastric inflammation in the mice from this experiment. As expected, mice immunized with *H. pylori* lysate plus CT exhibited enhanced gastritis after challenge (gastritis score 2.33±1.03 compared with naive challenged mice score of 1.0±0.82, p = 0.002 by ANOVA). Mice immunized with CTA1-UreB3T-DD did not exhibit enhanced gastritis (gastritis score 0.83±0.75, p = 0.68), perhaps reflecting the lower degree of protection when compared to mice immunized with bacterial lysate plus CT.

**Figure 4:**
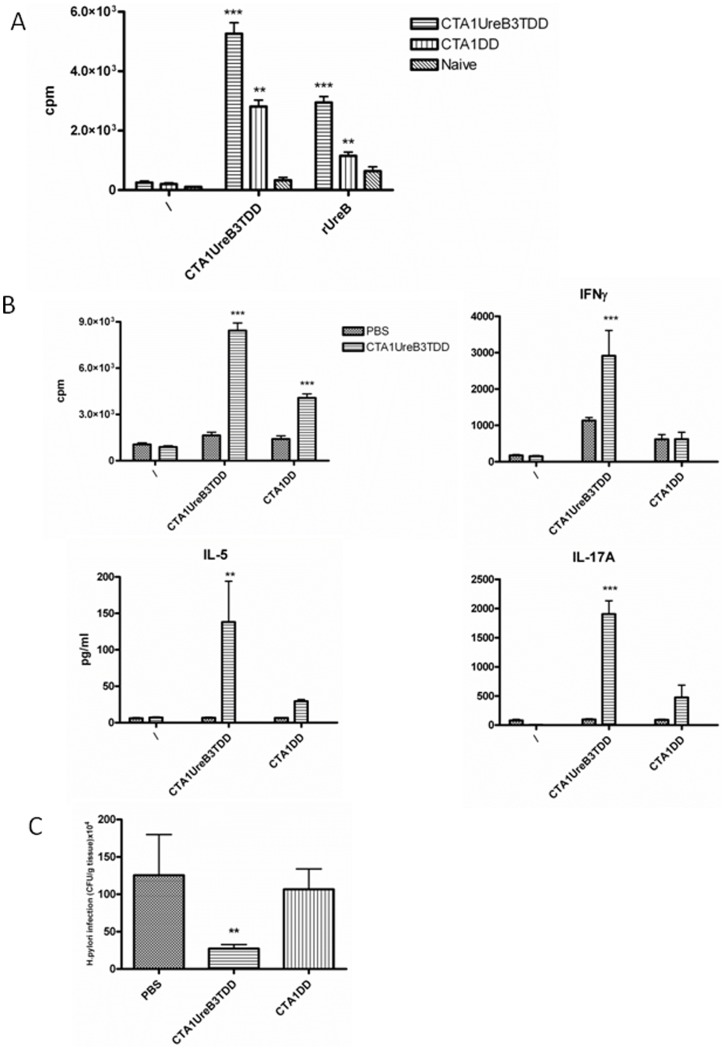
Mice (n = 5) were immunized once i.n with CTA1-UreB3T-DD or CTA1-DD alone and 10 days after the immunization spleen CD4 T cells were enriched using AutoMACS and cultured together with irradiated splenocytes from naïve nu/nu mice (T cell deficient) in the absence (\) or presence of CTA1-UreB3T-DD or rUreB at 1 µM and 10 µg/ml, respectively (A). Next, groups of 8 or 10 Balb/c mice were left unimmunized (PBS) or were immunized intranasally 4 times with 5 µg/dose of CTA1-UreB3T-DD (B–C) or 5 µg/dose CTA1-DD (C). Four weeks after completion of immunization a separate group of 3 naïve mice were left unchallenged and all other mice were challenged twice over three days with ca 2×10^7^ cfu of *H. pylori* SS-1 and 7 weeks later mice were killed and recall splenic T cell proliferation (B) and splenic whole cell recall responses of IL-5, IFNγ and IL-17A were determined in unstimulated (\) or stimulated (CTA1-UreB3T-DD (1 µM) or CTA1-DD (1 µM)) culture supernatants (B) and gastric bacterial colonization was assessed with *H. pylori* in cfu/g of stomach tissue (C). A is representative of 2 separate experiments while B–C are representive data from at least 5 identical experiments giving similar results. ANOVA was used for statistical analysis : * p<0.05, ** p<0.01 and ***p<0.001.

To verify that our CTA1-UreB3T-DD construct stimulated UreB-specific CD4 T cells we sorted CD4 T cells from the spleens of CTA1-UreB3T-DD or CTA1-DD-immunized or unimmunized mice by AutoMacs (>96% purity) and cultured these cells together with irradiated antigen-presenting cells (nu/nu splenocytes) and CTA1-UreB3T-DD or rUreB (Fig. 4A). We found that rUreB stimulated significant CD4 T cell proliferation in mice immunized i.n with the CTA1-UreB3T-DD construct, while CD4 T cells from CTA1-DD or unimmunized mice did not respond to recall rUreB (Fig. 4A). Moreover, the response to *in vitro* stimulation by CTA1-UreB3T-DD was stronger in mice immunized with the whole molecule than it was in mice immunized with CTA1-DD, without the UreB237–251 peptide. This indicated that UreB237–251, indeed, stimulated an UreB-specific CD4 T cell response (Fig. 4A). In fact, in 4 of 5 experiments, which included the CTA1-UreB3T-DD fusion protein, i.n immunization induced CD4 T cell responses that were associated with a 3–8 fold reduction in bacterial load as opposed to *H. pylori* challenge of unimmunized controls (not shown). The results from one of these experiments are shown in Figure 4. In this experiment immunization with CTA1-UreB3T-DD resulted in elevated levels of spleen cell recall proliferation, and in splenic IL-5, IFNγ and IL-17 cytokine production, as well as a 5-fold reduction in bacterial cfu after challenge. (Fig. 4B–C). As expected, i.n immunizations with CTA1-DD, lacking the UreBT-epitope, failed to stimulate anti-bacterial protection (Fig. 4C). Noteworthy, the recall responses to CTA1-UreB3T-DD, as opposed to those after CTA1-DD alone, which did not contain the UreB237–251 peptide, confirmed a significant CD4 T cell response against the UreB237–251-epitope (Fig. 4A–B). By contrast, immunizations with a mutant, enzymatically inactive, CTA1R7K-UreB3T-DD construct failed to stimulate protection or CD4 T cell responses, in agreement with our results from previous studies using inactive mutants (not shown) [29].

**Figure 5:**
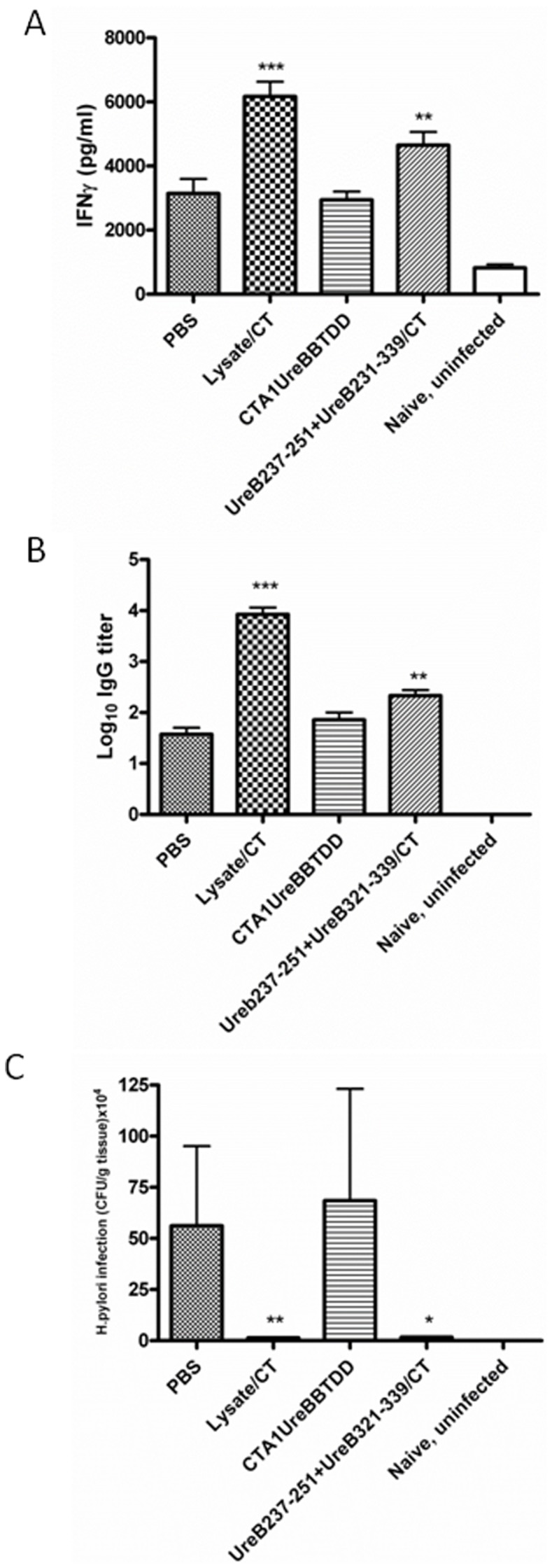
Groups of 6 or 7 Balb/c mice were left unimmunized (PBS) or were immunized intranasally 4 times with *H. Pylori* lysate+5 µg of CT adjuvant, CTA1-UreB T237–251/B 321–339-DD (a fusion protein containing 1 copy of the T epitope (UreB 237–251)+1 copy of the B epitope (B321–339)), UreB 237–251 alone+5 µg of CT adjuvant, or a mixture of UreB 237–251 and UreB B321–339+5 µg of CT adjuvant. Four weeks after completion of immunization a separate group of 3 naïve mice (Naïve) were left unchallenged and all other mice were challenged twice over three days with ca 2×10^7^ cfu of *H. pylori* SS-1 and 7 weeks later mice were killed and recall splenic IFNγ responses in supernatants from lysate-stimulated cultures with unstimulated background controls subtracted (A), serum IgG anti-*H. pylori* antibody responses (B) and gastric *H. pylori* cfu/g of stomach tissue (C) were determined. These are representive data from 2 identical experiments giving similar results. ANOVA was used for statistical analysis : *p<0.05, **p<0.01 and ***p<0.001.

These results, along with those in Figure 3, showing that a mixture of UreB237–251 and UreB321–339 (B cell epitope) plus CT-adjuvant yielded an enhanced IFNγ response and a nearly significant reduction in bacterial load, encouraged us to generate a CTA1-DD fusion protein, which contained a single copy each of both the T and B epitopes. However, in two experiments this CTA1-UreBBT-DD fusion protein failed to achieve any reduction in gastric *H. pylori* load, even though the non-fusion protein mixture of the two peptides plus CT did result in a slightly elevated IFNγ recall response and a reduction in bacterial load comparable to lysate in one of these experiments, as shown in Figure 5. Thus, we concluded that the fusion protein with 3 copies of the T cell epitope UreB237–251 was a more effective vaccine than a similar fusion protein with only a single copy of UreB237–251 (which also included a single copy of the B cell epitope UreB321–339) and significantly more effective than soluble peptide plus CT.

**Figure 6:**
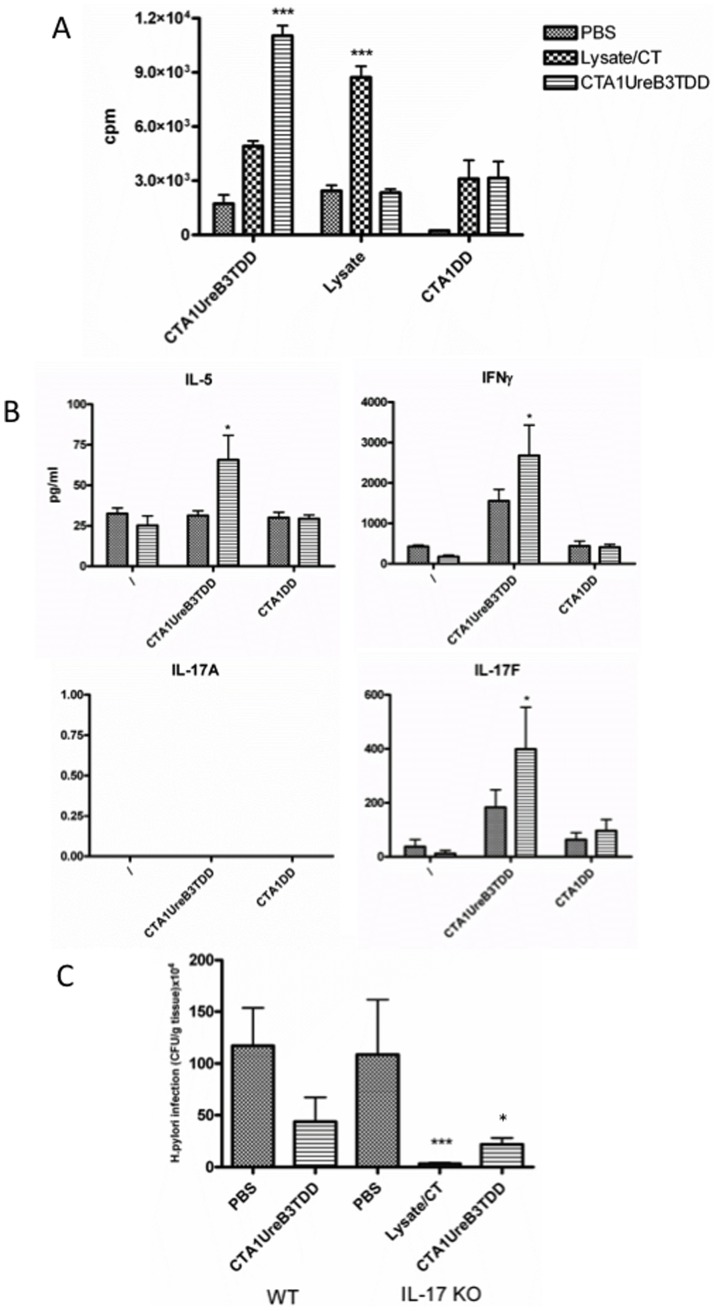
Groups of 8 or 10 wild-type (Balb/c) or IL-17A-deficient mice (H-2^d^ background) under SPF conditions were left unimmunized (PBS) or were immunized intranasally 4 times with 5 µg/dose of CTA1-UreB3T-DD or *H. Pylori* lysate+5 µg of CT adjuvant (IL-17KO only). Four weeks after completion of immunization a separate group of 3 naïve mice were left unchallenged and all other mice were challenged twice over three days with ca 2×10^7^ cfu of *H. pylori* SS-1 and 7 weeks later mice were killed and recall splenic T cell proliferation (A) and IL-5, IFNγ, IL-17F or IL-17A in culture supernatants were assessed after no stimulation (\) or stimulation *in vitro* with lysate, CTA1DD (1 µM) or CTA1UreB3TDD (1 µM) following i.n immunizations of IL-17KO mice, as indicated (B) and gastric *H. pylori* bacterial colonization as cfu/g of stomach tissue (C) was determined in both wild-type (WT) and IL-17KO mice. This is one representive experiment of three with IL-17 KO mice and one experiment comparing also with WT mice (C). ANOVA was used for statistical analysis: * p<0.05, **p<0.01 and ***p<0.001.

Because recent publications have investigated a possible role for IL-17 in vaccine-mediated immunity and protection against *H. pylori* infection [37], [38], [39] and our results with CTA1-UreB3T-DD showed enhanced priming of IL-17A producing T cells following i.n immunizations (Fig. 4B) we investigated the protective effect of CTA1-UreB3T-DD in IL-17A-deficient and wild-type Balb/c mice [31]. Strikingly, CTA1-UreB3T-DD was equally effective in IL-17A-deficient as compared to WT mice with regard to protection and also effectively primed CD4 T cells in IL-17A-deficient mice (Fig. 6A–C). Hence, when immunized IL-17A-deficient mice were challenged with live *H. pylori* they showed a 5-fold reduction in bacterial load compared with mice immunized with CTA1-DD, without the UreB peptide, or given PBS only (Figure 6C). Again supernatants stimulated with CTA1-UreB3T-DD and CTA1-DD, respectively, differed with regard to the cytokine responses, indicating that CD4 T cells recognizing the UreB237–251 peptide were stimulated by CTA1-UreB3T-DD (Fig. 6B). As expected, CD4 T cells from mice immunized i.n with CTA1-UreB3T-DD had much stronger recall responses to the fusion protein than against bacterial lysate, whereas lysate immunized mice had fewer CD4 T cells that could respond to the fusion protein (Fig. 6A). The results also showed that, as expected, no IL-17A was produced in CD4 T cell recall responses in IL-17A deficient mice, while supernatants exhibited significantly elevated levels of IL-5, IFNγ and IL-17F when taken from cultures of immunized mice (Figure 6B).

## Discussion and Conclusions

The unexpectedly slow progress in anti-*Helicobacter* vaccine research can be ascribed to a poor understanding of the immunopathology and immune evasion mechanisms employed by *H. pylori*, subsequent waning pharmaceutical/vaccine company enthusiasm, as well as the lack of safe and effective mucosal vaccine formulations and delivery systems/adjuvants [6], [40]. Therefore, unravelling the mechanisms of *Helicobacter*-specific protective immunity using the mouse model is still highly relevant and much needed, even from an industrial perspective [41]. The poor outcome of clinical vaccine trials in the past has certainly also contributed to a declining interest in *H. pylori*-vaccine development. Apart from whole killed bacteria, also urease or combinations of CagA, VacA and neutrophil-activating protein (NAP) have been tested in human clinical trials with quite poor outcome [42]. Although parenteral vaccination has been reported to induce memory T cells specific for *H. pylori*, most experimental studies have clearly documented that mucosal immunizations are more effective at stimulating *Helicobacter*-specific protective immunity in the gastric mucosa. Particularly interesting are vaccines that effectively have stimulated *H. pylori*-specific CD4 T cells and the successful immunizations using subcomponent vaccines in mouse models [18].

While whole bacterial cell lysate approaches to vaccine-development has been attempted for a long time these are unrealistic for commercial production and more and more subcomponent vaccines have been described. Antioxidants have attracted interest as subcomponent vaccines and catalase, thiolperoxidase or superoxide dismutase, all proteins involved in bacterial colonization and survival, are three candidates to potentially be included in a vaccine [43]. Also, Urease B (UreB) has been found to be a strong vaccine candidate and it has provided excellent results in experimental models. Indeed, recent attempts to immunize with whole recombinant UreB and a truncated form of neuraminyllactose-binding hemagglutinin (HpaA), a *H. pylori*-specific lipoprotein, induced strong immune protection comparable to that of whole cell lysate [44]. Attempts to develop UreB peptide-based immunization protocols have also been reported in mice and immunodominant epitopes in *H. pylori*-infected subjects have recently been reported [18], [45], [46].

We have developed a peptide-based CTA1-DD adjuvanted *H. pylori* vaccine for mucosal administrations. We hypothesized that by exploring the CTA1-DD adjuvant as a carrier and immunomodulator of UreB-specific MHC class II-restricted peptides, we could achieve strong protection against infection, and with minimal amounts of peptide [18], [28], [29]. For comparison we initially also used rather large doses of peptide alone together with CT holotoxin, as the adjuvant, to generate a significant level of protection against a live *H. pylori* challenge infection. We found that protection in the range of 3–8-fold reduction of bacterial colonization was effectively achieved with a fusion protein that carried the UreB 327–251 peptide, in 3 tandem repeats, while a single epitope or just a single linear B cell epitope of UreB 321–339, was without protective effect following 4 i.n immunizations. Noteworthy, the assessment of protection following immunizations and challenge was 3 or 7 weeks, a time difference that could have contributed to the variablity in protective capacity of CTA1-UreB3T-DD (see Materials & methods). However, previous studies have clearly shown only very small differences in bacterial load in immunized and protected mice at 3 and 7 weeks [33], [34]. Interestingly, also i.n immunizations with the constructs gave comparable protection in IL-17A-deficient mice, indicating that IL-17A was not absolutely required to elicit a protective immune response. This result confirms our previous published report which utilized an *H. pylori* whole cell lysate vaccine [37], but it is in contrast to some other reports which used anti-IL17 monoclonal antibodies to neutralize IL-17A during the effector phase after *H. pylori* challenge [38], [39]. In our present results protection in IL-17 null mice correlated with IFNγ levels, while in wild-type (WT) mice also IL-17A and IL-5 levels were strongly up-regulated in CD4 T cells mediating protective immunity, as compared to CD4 T cell responses in unprotected mice. Thus the role of IL-17A or IL-17F in protective immunity remains unclear, but could be related to the well known role of IL-17A/F in promoting the induction of neutrophil chemokines, which could be substituted for by other neutrophil chemotactic factors, as discussed in our previous publication [37]. Nevertheless, our study clearly suggests that in the absence of IL-17A and Th17 cells, Th1 cells may compensate and provide significant protection following vaccination. This is in agreement with recent results exploring vaccine-induced protection in IL23 p19-deficient mice and the fact that IL-12 i.p treatment of *H. pylori*-infected mice promoted eradication of infection even without prior vaccination [47]. In total, our study clearly demonstrates that it is feasible to design and develop novel mucosal vaccines against *H. pylori* infections by exploring the CTA1-DD adjuvant platform and selected CD4 T cell epitopes. In conclusion, we have provided evidence for the rational design of an effective mucosal subcomponent vaccine against *H. pylori* infection based on well selected protective epitopes from relevant antigens incorporated into the CTA1-DD adjuvant platform.
